# Minimally invasive surgical management of mid- to distal esophageal diverticula: a large single-center review

**DOI:** 10.1016/j.gassur.2026.102427

**Published:** 2026-04-14

**Authors:** Marissa A. Matto, James D. Luketich, Ryan M. Levy, Gabriella Lloyd, Omar Awais, Tadeusz Witek, Neil A. Christie, Navid Ajabshir, Evan T. Alicuben

**Affiliations:** Division of Thoracic Surgery, Department of Cardiothoracic Surgery, University of Pittsburgh Medical Center, Pittsburgh, Pennsylvania, United States

**Keywords:** Benign esophageal disorders, Epiphrenic diverticulum, Esophageal diverticulum, Minimally invasive surgery

## Abstract

**Purpose:**

This study aimed to investigate the outcomes of minimally invasive surgery for mid- to distal thoracic esophageal diverticula (TED).

**Methods:**

This was a retrospective review of patients who underwent surgery for symptomatic TED. Patients with Zenker diverticulum were excluded. Demographic information and operative details were recorded. Dysphagia score was defined using the Knyrim scoring system (0 = no dysphagia, 1 = hard solids, 2 = soft solids, 3 = liquids, and 4 = saliva). The follow-up included clinical encounters, barium esophagram in all patients, and endoscopy in those who developed postoperative symptoms.

**Results:**

From September 2001 to September 2024, there were 91 patients with a median follow-up of 19 months. Of note, 79 patients (86.8%) presented with dysphagia. Achalasia was identified in 27 patients. Surgical approaches included thoracoscopy (58.2%), laparoscopy (31.9%), or both (8.8%). Myotomy was performed in 85.7% of patients. There were no perioperative mortalities. The median hospital length of stay was 6 days. Of note, 6 patients had an esophageal leak postoperatively, and 4 patients required intervention. Of the 26 patients with a residual or recurrent diverticulum, only 4 required reoperation. At the first postoperative visit, all patients reported an improvement in their dysphagia scores, and 88.6% of patients reported no dysphagia. At a median follow-up of 19 months, 30 patients reported some degree of dysphagia, but overall, the dysphagia scores remained significantly improved compared with those in the preoperative period (P < .001).

**Conclusion:**

Minimally invasive treatment of symptomatic, mid- to lower TED resulted in significant improvement in dysphagia scores and low morbidity.

## Graphical Abstract

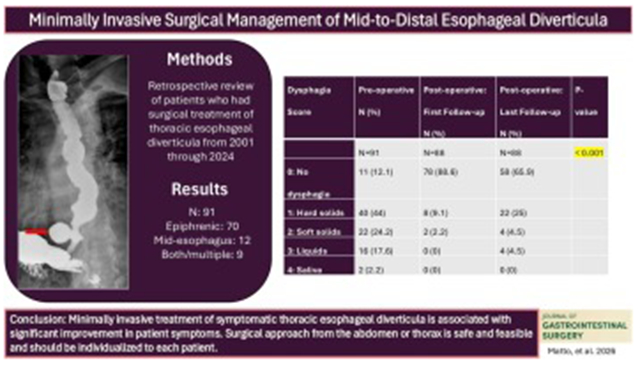

## Introduction

Mid- to lower esophageal diverticula are rare disorders of the esophagus, occurring in < 1% of the population [1]. Diverticula can be caused by traction secondary to adjacent inflammation in some cases. However, they are often associated with esophageal motility disorders and are most commonly pulsion diverticula. Epiphrenic diverticula are usually pulsion diverticula, and the distal margin is within 4 cm of the gastroesophageal junction, whereas mid-esophageal diverticula are usually traction related. These diverticula can be located at variable locations and frequently do not have a measurable manometric abnormality or distal narrowing [1].

Although there is agreement that surgical treatment of esophageal diverticula is recommended for symptomatic patients with large diverticula, there is some controversy on the approach and which procedures should be performed [1]. Historically, patients have been treated with an open transthoracic approach [2]. With advances in minimally invasive surgery (MIS), esophageal diverticula are now more often treated either laparoscopically or thoracoscopically [3]. Our institution reported the largest series of minimally invasive surgical management for thoracic esophageal diverticula (TED) with primary end points of 30-day mortality and other perioperative outcomes. The main conclusion was that MIS is safe and effective at an intermediate-term follow-up of 20.8 months [4]. With minimally invasive strategies becoming our standard approach, this study aimed to establish the outcomes of MIS across a larger series of patients with TED.

## Materials and methods

### Data collection and analysis

This was a retrospective review of patients who underwent esophageal diverticulum surgical treatment between September 2001 and September 2024. Patients with Zenker diverticulum were excluded from the study. This study was conducted under an institutional review board–approved protocol (approval number: STUDY20050010), and individual consent was waived. Demographic information, operative details, and postoperative outcomes were collected. The primary outcome of this study was a change in dysphagia scores. The dysphagia scores were based on the Knyrim scoring system, which is a modification of the Mellow and Pinkas score [5]. This scoring system was used for its simplicity, which allowed us to assign most patients a score based on review of medical documentation. Dysphagia was scored on a scale of 0 to 4 (Table 1). Pre- and postoperative dysphagia scores were analyzed using the Wilcoxon signed-rank test.

### Preoperative workup

**Figure 1.**
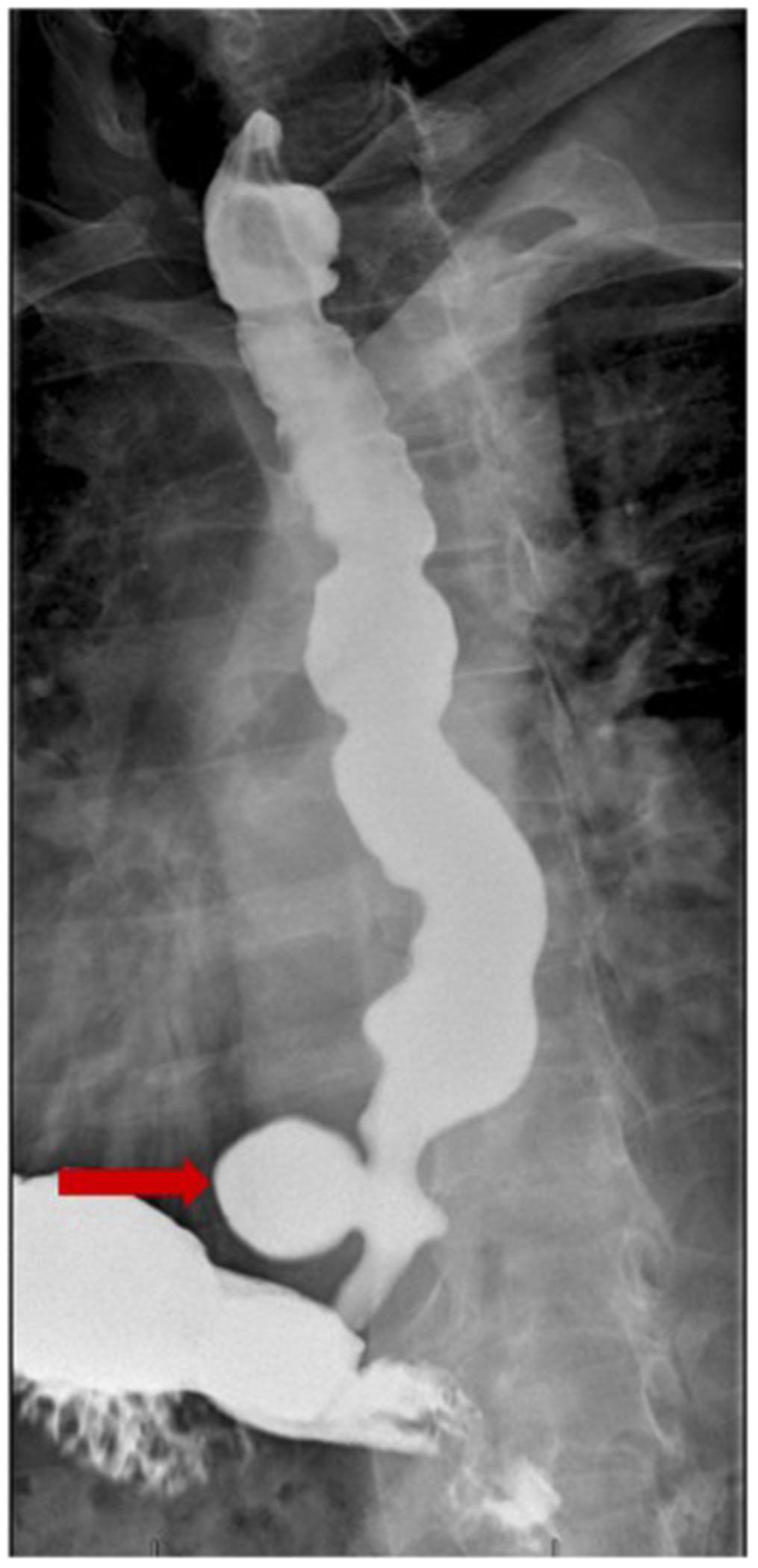
Barium esophagram demonstrating an epiphrenic diverticulum (red arrow) with a wide mouth and evidence of abnormal esophageal motility.

**Figure 2.**
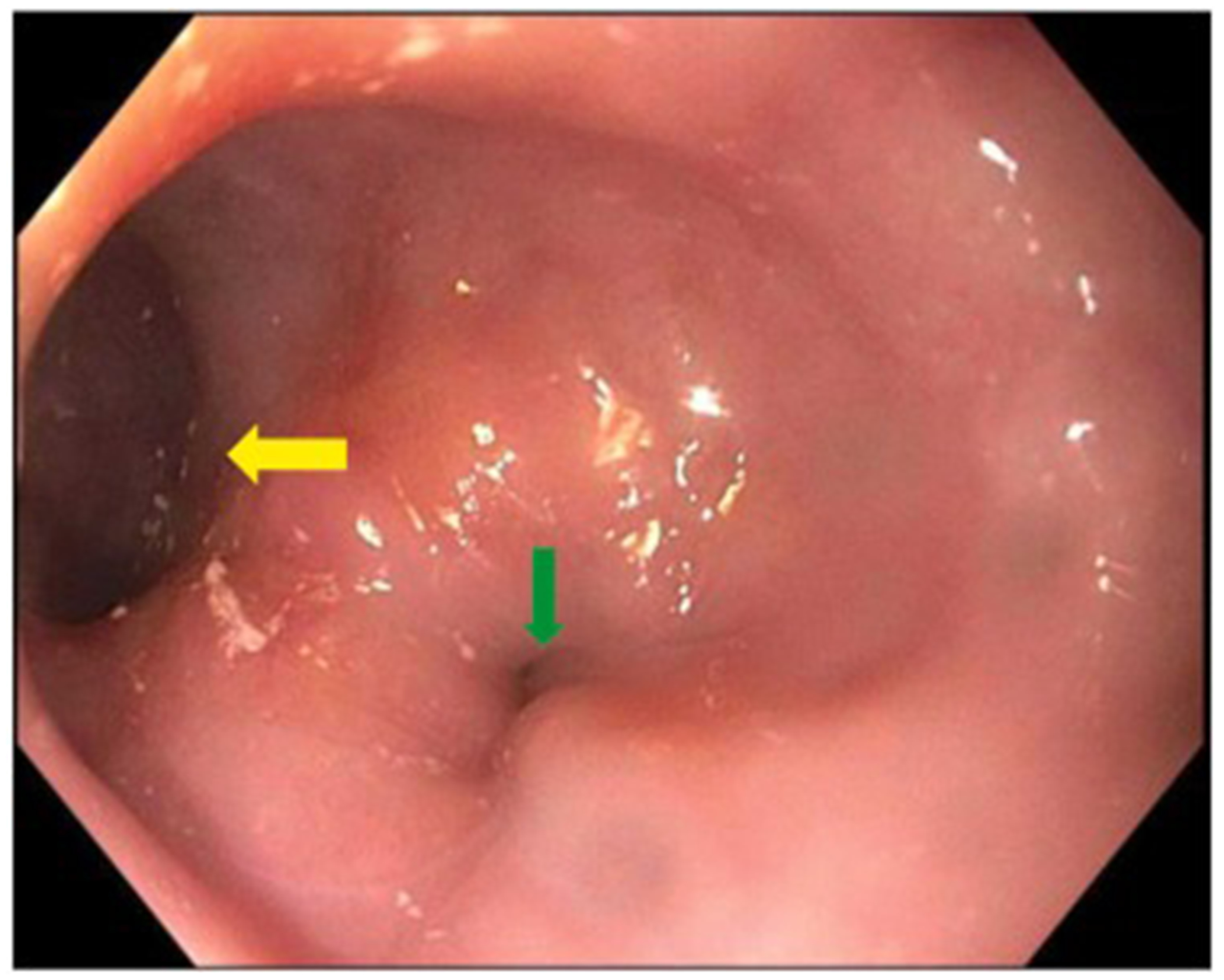
Esophagogastroduodenoscopy demonstrating an epiphrenic diverticulum (yellow arrow). The esophageal lumen can be seen in the center of the figure (green arrow).

Our general patient workup includes a barium swallow study, esophagogastroduodenoscopy (EGD), and manometry testing. Barium esophagram demonstrates the diverticulum size, location, and side of the esophagus from which the diverticulum arises (Fig. 1). It can also assist in the identification of abnormalities in motility. EGD is important for assessing the location of the diverticulum, the diameter of the neck, the presence of downstream narrowing, and concomitant esophageal pathology (Fig. 2). Manometry can be used to diagnose underlying motility disorders contributing to the formation of the diverticulum, which generally needs to be addressed during surgery.

### Operative technique

We previously published our operative technique [6]. The choice of operation depends on the surgeon and can be performed from the abdomen or chest. This decision largely depends on the location of the diverticulum and any additional planned operations. Heller myotomy and fundoplication are performed via an abdominal laparoscopic approach, whereas a myotomy and diverticulectomy for a midesophageal diverticulum necessitates a thoracic approach. An EGD should always be performed at the start of the operation to remove any retained food debris and measure the exact location and size of the diverticulum. The principles of diverticulectomy include preservation of the vagus nerves, dissection of the neck of the diverticulum, and use of the endoscope or bougie within the esophagus while stapling to avoid luminal narrowing. A myotomy is required for complete dissection of the base of the diverticulum. In some cases, the myotomy is closed after performing diverticulectomy. An additional myotomy may be performed, whereby the location is determined at the surgeon’s discretion and is performed either ipsilateral or contralateral to the diverticulum. In some cases, no further myotomy is performed, and these cases usually have no downstream narrowing or motor disorder. As many patients have an underlying esophageal motility disorder, myotomy is generally performed. If the myotomy is performed across the lower esophageal sphincter, a Dor fundoplication is commonly the antireflux operation of choice. In most cases, a surgical drain is placed near the diverticulectomy site.

Here, we defined an extended myotomy as a myotomy extending beyond the edges of the diverticulum or at a site on the esophagus different from the location of the diverticulum. A limited myotomy was defined as one that is at the site of the diverticulum and only extends to the edges of it. A myotomy closure was defined as a limited myotomy in which the longitudinal muscle is sutured over the staple line.

### Postoperative management

Postoperatively, the patient is kept nothing by mouth until a barium esophagram is performed on postoperative day 1 or 2. If the study is negative for leak, the diet is advanced from liquids to soft to regular over a 2-week period. Once a liquid diet is tolerated, the patient can be discharged. Patients are seen in the clinic post-operatively and then annually with an esophagram. Endoscopy is performed if abnormalities in the esophagram are observed or if the patients are symptomatic.

## Results

From September 2001 to September 2024, 91 patients were treated for esophageal diverticulum. Table 2 presents the demographic data and characteristics of the patients. The most common symptom was dysphagia (86.8%). Other common symptoms included regurgitation, heartburn, cough, and aspiration. Of note, 67 patients had documented manometry, and achalasia (40.3%) was the most common esophageal motility disorder identified, followed by ineffective esophageal motility (23.9%). The diverticulum was in an epiphrenic location in 76.9% of patients.

Operative and perioperative data are presented in Table 3. Of note, 58.2% of patients had thoracoscopy, 31.9% of patients had laparoscopy, and 8.8% had both thoracoscopy and laparoscopy. The median operative time was 306 min. Of note, 2 patients had an urgent inpatient surgery, and 1 patient had an emergent operation, each with a unique indication. One was due to a giant epiphrenic diverticulum with an associated bezoar. Moreover, 1 patient presented with severe aspiration and dehydration leading to acute renal failure. After treatment of aspiration and renal failure, the patient underwent surgery during the same admission. The emergent patient presented with a giant paraesophageal hernia, large diverticulum, and gastric volvulus with evidence of ischemia. Conversion to open thoracotomy or laparotomy occurred in 5 patients, all of whom had adhesions and inability to progress.

Of note, 58 patients received a diverticulectomy with myotomy. Of these patients, 46 had an extended myotomy, either at the site of the diverticulum or somewhere else on the esophagus. Moreover, 12 patients had a limited myotomy only extending to the edges of the diverticulum. The location of the myotomy was ipsilateral to the diverticulum in 41 patients, anterior (Heller) in 29 patients, and contralateral to the diverticulum in 7 patients. Of the remaining patients, 19 underwent diverticulectomy, extended myotomy, and fundoplication. Moreover, 8 patients had a diverticulectomy with myotomy closure. Of note, 1 patient had a diverticulectomy alone without myotomy. This patient had a midesophageal traction diverticulum that developed into a bronchoesophageal fistula. Moreover, 1 patient underwent an endoscopic approach.

Postoperative complications are presented in Table 3. The most common postoperative complication was atrial fibrillation (9.9%). Of note, 6 patients were diagnosed with a staple line leak, and 4 patients required intervention. No association was observed between the incidence of leak and the type of operation performed: 2 had diverticulectomy with extended myotomy, 2 had diverticulectomy with myotomy closure, 1 had diverticulectomy with limited myotomy, and 1 had diverticulectomy alone. The 2 patients who did not require an intervention were managed with the drain placed during the initial operation. Both patients presented with a sub-clinical leak that was seen radiographically on barium swallow. They were continued on a soft diet with the drain in place. Of the 4 patients managed with an intervention, 3 were managed with surgery. However, 1 patient presented with fever and leukocytosis. This patient required an esophageal exclusion due to a history of unknown bariatric surgery (suspected to be a vertical sleeve gastrectomy) and evidence of gastric ischemia. Of note, 2 patients had contained leaks with mediastinal abscesses but were minimally symptomatic. Of the 2 patients, one required thoracotomy and primary repair of the perforation, which was sutured in layers, and the other had computed tomography–guided percutaneous drain placement and a laparoscopic gastrostomy tube for feeding access. Finally, the patient who did not have a reoperation was asymptomatic and had a punctate leak on barium esophagram. This patient was treated with an esophageal stent, which was removed after 4 weeks, and the diet was advanced uneventfully.

Table 4 presents the follow-up comparison of dysphagia scores. At the initial follow-up, there was a significant improvement in dysphagia symptoms in all patients. Moreover, 88.6% of patients reported no dysphagia. At a median follow-up of 19 months, 65.9% of patients remained dysphagia-free, and the remaining patients had significantly improved dysphagia compared with preoperative scores (*P* < .001). Of note, 19 patients were lost to follow-up after the first postoperative visit.

Of note, 6 patients ultimately underwent esophagectomy at a median time of 6.5 months (IQR, 0.0–33.0). Of these 6 patients, 4 had an underlying esophageal motility disorder that progressed to recalcitrant symptoms and required esophagectomy. Of note, 1 patient had normal esophageal motility and required esophagectomy for recurrent diverticula after 2 failed diverticulectomies. The first diverticulectomy was performed at an outside hospital and required re-excision 3 years later, which was performed at our institution. During the second operation, a myotomy was performed along with the diverticulectomy. During the completion of the endoscopy, a second, more distal diverticulum was identified but was not resected due to the tenuous and tortuous state of the esophagus. Finally, 1 patient had a history of Roux-en-Y gastric bypass and an epiphrenic diverticulum proximal to the gastrojejunal anastomosis. The initial operation was a repair of the diverticulum by imbricating the gastric pouch over it. This ultimately required distal esophagogastrectomy with esophagojejunal anastomosis due to a stricture and dysphagia with no evidence of a recurrent diverticulum.

## Discussion

Minimally invasive surgical intervention for symptomatic esophageal diverticula resulted in an initial improvement in dysphagia scores in all patients, with a low morbidity in this large group of patients. The surgical approach was tailored to the location of the diverticulum and the presence or absence of an associated motor disorder or downstream narrowing.

MIS has been associated with decreased postoperative pain and shorter hospital lengths of stay than traditional open approaches [7]. In both open and minimally invasive approaches to esophageal diverticula, symptomatic improvement has been reported in most patients using various surgical approaches [2,4,8–12]. In the 2 largest studies of open thoracic approaches [2,8], which included 17 and 35 patients, respectively, the median length of stay was 7 days. This is comparable to our study, which demonstrated a median length of stay of 6 days. Tapias et al. [2] reported an overall morbidity rate of 35.5%. However, when considering Clavien-Dindo complications ≥ IIIb, our study had a complication rate of 13.2% compared with 19.4% after open intervention. These differences indicate that minimally invasive approaches are beneficial in decreasing severe complications. Here, the leak rate that required reoperation was 3.3% compared with leaks in the open series at 5.7% and 0.0% [2,8]. Because of the limited number of leaks, it is difficult to determine whether MIS offers an advantage in this regard. Most of these leaks occurred earlier in the series, and therefore, only 1 case reflects more modern approaches to leak treatment, such as stent placement. In addition, improvements in surgeon experience and stapler technology may contribute to the lack of staple line leaks in recent years.

Other studies have shown that 2% to 6% of patients with achalasia eventually need esophagectomy [13,14]. Of note, 1 study demonstrated differences in esophagectomy rate in patients with achalasia who have undergone previous esophageal surgery (6%) compared with those who had no previous esophageal surgery at 2% [14]. Here, 6.6% of patients with diverticula ultimately required esophagectomy, which is consistent with patients with achalasia who have undergone previous esophageal surgical procedures. Although the esophagectomy rate seems high, we believe that the formation of subsequent esophageal diverticula indicates a weakened esophagus and may be a risk factor for progression to end-stage symptoms. Furthermore, not all previous reports included significant follow-up. Thus, the ultimate disposition of these patients is not known.

To the best of our knowledge, this is the largest known minimally invasive series to date. In a previous study from our institution, the leak rate that required reoperation or procedural intervention was 7% [4]. In this current study, the leak rate requiring either reoperation or EGD intervention was 4.3%. It is likely that increased experience in using minimally invasive techniques, in conjunction with improved perioperative care, contributed to the improvement in clinically significant leak rates. Compared with other studies analyzing minimally invasive approaches, our leak rate was similar, and the median hospital length of stay was slightly superior: 6.5 days (3.3%) [12] and 7.0 days (5.0%) [11].

An alternative approach to standard surgical techniques is diverticular peroral endoscopic myotomy (D-POEM), which was performed in 1 patient in our series. This technique has been shown to have high rates of symptom improvement [15,16]. There are no current recommendations indicating which patients would benefit from D-POEM over traditional surgical intervention. Longer-term studies addressing symptom recurrence or worsening of residual diverticula are not yet available. The patient in our study had a history of achalasia with a small diverticulum and had no complications. This patient demonstrated complete resolution of dysphagia with 22 months of follow-up.

**Figure 3.**
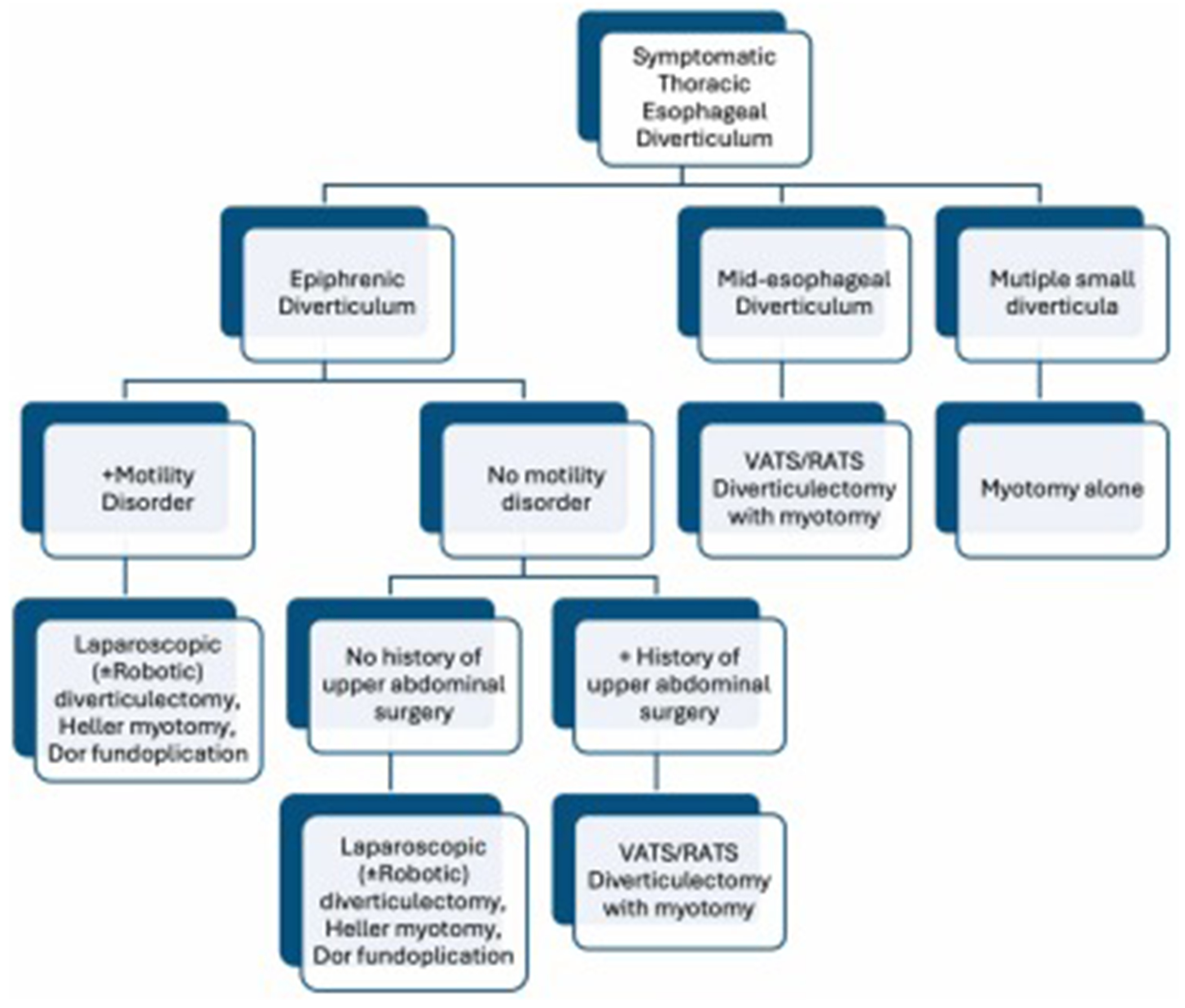
Clinical pathway for surgical treatment of thoracic esophageal diverticula. RATS, robotic-assisted thoracoscopic surgery; VATS, video-assisted thoracoscopic surgery.

Although diverticulum location frequently dictates the surgical approach (ie, thoracoscopic vs laparoscopic), there are instances in which either is a viable option. In these patients, we prefer a right thoracoscopic approach. This enables exposure to both sides of the esophagus and provides better visualization of the esophagus proximal to the diverticulum than a laparoscopic approach. Given our experience in performing many esophageal procedures from right thoracoscopy, we also approach a left-facing diverticulum through the right thorax. With mobilization of the esophagus, the diverticulum can be rotated for both myotomy and resection. Fig. 3 proposes an algorithm for the surgical treatment of a thoracic esophageal diverticulum.

Here, there were several differences in the surgical procedures performed. Although most patients underwent diverticulectomy and extended myotomy, some patients underwent diverticulectomy with limited myotomy or myotomy closure. Patients who underwent myotomy alone had smaller or multiple diverticula, and therefore, their symptom improvement could only be attributed to the myotomy. In other patients with a large diverticulum, especially those with a wide neck that holds retained food or liquid, with no distal narrowing or evidence of a tight lower esophageal sphincter, it is reasonable to assume that diverticulectomy alone will yield most of the symptom improvement. In most patients, we recommend a diverticulectomy with an extended myotomy. This myotomy can be performed at the location of the diverticulum with distal extension of the myotomy, or the diverticulum site can be oversewn and the myotomy performed on the contralateral aspect of the esophagus. If the myotomy crosses the gastroesophageal junction, we recommend a Dor fundoplication to prevent reflux. Ultimately, treatment of this rare condition requires an individualized, patient-centered approach. Some patients in our series with a diverticulum had no evidence of downstream narrowing or motor disorder. In those instances, we routinely included a myotomy. Many patients with esophageal diverticula have an associated motor disorder, which necessitates myotomy. Therefore, we recommend performing manometry in all patients presenting with this disorder, especially those with an epiphrenic diverticulum. Of note, 24 patients in our series did not have documented preoperative manometry testing. Many of these patients were treated before 2010, had midesophageal diverticulum, or had an obvious clinical reason for the formation of their diverticulum (eg, tight fundoplication).

Study limitations include the relatively short median follow-up and its retrospective nature. Although the range of follow-up was large (1–226 months), several patients were lost to follow-up, which precluded longer median follow-up. Because of its retrospective design, this study is subject to selection bias and loss of data due to inconsistent medical documentation. We hope to improve on this by using validated quality-of-life surveys to characterize patient symptoms. Further comparative studies could characterize the benefits of certain approaches with increased use of robotic and complex endoscopic technology. Finally, these operations were performed at a specialized center with a high volume of esophageal surgery, limiting the generalizability of these data to less specialized institutions.

## Conclusion

Our study is the largest series reporting on the surgical treatment of mid- to distal esophageal diverticula. MIS is a safe approach with significant improvement in patient symptoms. Both laparoscopic and thoracoscopic approaches are feasible and can be chosen based on the location of the diverticulum and the surgeon’s preference.

## Figures and Tables

**Table 1:** Dysphagia scoring system.

Dysphagia score	Definition
0	No dysphagia
1	Able to swallow some hard solids
2	Able to swallow some soft solids
3	Able to swallow some liquids
4	Unable to swallow saliva

**Table 2:** Demographics and presenting characteristics.

Variable	n (%) or median (IQR)
N	91
Female	56 (61.5)
Male	35 (38.5)
Age, y	69 (23–96)
BMI, kg/m2	26.0 (15.4–47.5)
Smoking history: former or current	39 (42.9)
Charlson Comorbidity Index score	1 (0-11)
Manometry	67 (73.6)
Achalasia	27 (40.3)^a^
Ineffective esophageal motility	16 (23.9)^a^
Normal	9 (13.4)^a^
Hypertensive lower esophageal sphincter	8 (11.9)^a^
Diffuse esophageal spasm	6 (9.0)^a^
Nutcracker esophagus	1 (1.5)^a^
Surgical history: upper abdominal	
Cholecystectomy	17 (18.7)
Diverticulectomy with myotomy	2 (2.2)
Diverticulectomy with myotomy closure	2 (2.2)
Hiatal hernia repair with fundoplication	2 (2.2)
Roux-en-Y gastric bypass	1 (1.1)
Sleeve gastrectomy	1 (1.1)
Preoperative symptoms	
Dysphagia	79 (86.8)
Regurgitation	59 (64.8)
Heartburn	48 (52.7)
Cough	24 (26.4)
Epigastric or chest pain	20 (22.0)
Aspiration	18 (19.8)
Weight loss	17 (18.7)
Recurrent pneumonia	14 (15.4)
Abdominal pain	12 (13.2)
Location of diverticulum	
Epiphrenic	70 (76.9)
Midesophagus	12 (13.2)
Both or multiple diverticula	9 (9.9)

BMI, body mass index.

a Percentage calculation based on the number of patients who had manometry testing.

**Table 3:** Operative data and postoperative complications.

Variable	n (%) or median (IQR)
Surgical approach	
Laparoscopy	29 (31.9)
Thoracoscopy	53 (58.2)
Laparoscopic/thoracoscopic	8 (8.8)
Endoscopy	1 (1.1)
Conversion to open approach	5 (5.5)
Urgent or emergent	3 (3.3)
Operative time, min	306 (105–944)
Operation performed	
Diverticulectomy with extended myotomy	46 (50.5)
Diverticulectomy with limited myotomy	12 (13.1)
Diverticulectomy/extended myotomy/fundoplication	19 (20.9)
Diverticulectomy with myotomy closure	8 (8.8)
Diverticulectomy/fundoplication	2 (2.2)
Diverticulectomy only	2 (2.2)
Myotomy only	1 (1.1)
Other	1 (1.1)
Myotomy location	
Ipsilateral to diverticulum	41 (52.6)^a^
Anterior/Heller	29 (37.2)^a^
Contralateral to diverticulum	7 (9.0)^a^
Bougie or endoscope used for stapling	77 (86.5)^b^
Hospital length of stay, d	6 (1–57)
30-d mortality	0 (0.0)
30-d morbidity	33 (36.3)
Clavien-Dindo grade I, II, or IIa	21 (23.1)
Clavien-Dindo grade IIIb or IVa	12 (13.2)
Atrial fibrillation	9 (9.9)
pneumonia	8 (8.7)
Pleural effusion requiring chest tube	6 (6.6)
Leak requiring reoperation	3 (3.3)
Readmission < 30 d	7 (7.7)
Recurrent diverticulum	14 (15.4)
Residual diverticulum	12 (13.2)
Recurrent/residual required redo	4 (15.4)^c^
Esophagectomy	6 (6.6)

a Only Patients who had a myotomy were included in this percentage, n = 78.

b Patients who did not receive diverticulectomy were excluded in this percentage, n = 89.

c Percentage based on number of patients with recurrent/residual diverticulum, n = 26.

**Table 4:** Preoperative and postoperative dysphagia scores.

Dysphagia score	Preoperative, n (%)	Postoperative: first follow-up, n (%)	Postoperative: last follow-up, n (%)	*P* value^a^
	n = 91	n = 88	n = 88	< .001
0: no dysphagia	11 (12.1)	78 (88.6)	58 (65.9)	
1: hard solids	40 (44.0)	8 (9.1)	22 (25.0)	
2: soft solids	22 (24.2)	2 (2.2)	4 (4.5)	
3: liquids	16 (17.6)	0 (0.0)	4 (4.5)	
4: saliva	2 (2.2)	0 (0.0)	0 (0.0)	

a Wilcoxon signed-rank test.
